# Job Satisfaction as a Predictor of Emotional Labor Among Nurses: A Cross-Sectional Study

**DOI:** 10.1155/jonm/8062261

**Published:** 2025-08-07

**Authors:** Wejdan Shaqiqi

**Affiliations:** ^1^College of Nursing, King Saud bin Abdulaziz University for Health Sciences, Riyadh, Saudi Arabia; ^2^King Abdullah International Medical Research Center, Riyadh, Saudi Arabia

**Keywords:** deep acting, emotional labor, satisfaction, surface acting

## Abstract

**Background:** Emotional labor, which plays a significant role in nursing, can be influenced by job satisfaction. High levels of nurses' job satisfaction can lead to appropriate emotional engagement and demonstration, positively impacting patient care and satisfaction. Despite its significance, most studies have examined nurses' job satisfaction as an outcome of emotional labor rather than as a predictor.

**Aim:** To assess the influence of nurses' job satisfaction on their emotional labor.

**Method:** The study used a cross-sectional, correlational, descriptive design. A convenience sample of 125 bedside nurses was recruited from a public hospital in Riyadh, Saudi Arabia. The data were collected using the Emotional Labor Scale for Nurses and the Michigan Organizational Assessment Questionnaire Job Satisfaction scale from June to August 2024.

**Result:** The emotional labor of nurses was high (*M* = 58.04, SD ± 9.44); emotional control effort in the profession was the most frequent strategy used (*M* = 28.65, SD ± 5.09), followed by patient-focused emotional suppression (*M* = 16.15, SD ± 3.82), and the least common was emotional pretense by norms (*M* = 13.23, SD ± 2.89). Nurses' job satisfaction was positively correlated with their emotional labor (*r* = 0.220, *p*=0.017) and emotional control effort in the profession (*r* = 0.258, *p*=0.004), and it influenced their EL (*β* = 0.22, *p*=0.014).

**Conclusion:** Our findings reveal that nurses experience high levels of emotional labor when interacting with patients. Job satisfaction can influence how nurses experience and manage emotional labor in the workplace.

**Implications:** The study recommends that policymakers enhance job satisfaction among nurses to support the more effective and appropriate use of emotional labor strategies, thereby mitigating negative impacts on both the nurses and the organization.

## 1. Background

The concept of emotional labor (EL) was first introduced by Hochschild in 1983 to refer to the process in which the employee manages his feelings and displays expression when interacting with a client, colleagues, and superiors to fulfill their job duties and cultural expectations [1]. Hochschild suggested that EL is performed through surface and deep acting. Surface acting involves displaying appropriate emotions at work that are not genuinely felt and suppressing or hiding one's inner feelings, whereas deep acting involves trying to evoke or shape one's inner feelings to match the emotions that one is required to display. EL was introduced into nursing by Smith in 1991 [2]. Since then, the number of studies of EL among nurses has significantly increased, confirming that it is an integral skill of the nursing profession.

EL is a core component of nursing work. According to Smith [2], the heart of nursing is caring, which involves emotion. Nursing requires not only cognitive and physical labor but also EL to display and control one's emotions while providing care to patients. Nurses routinely engage in EL in their everyday interactions with patients, families, colleagues, and supervisors [2]. This includes alleviating patients' pain and worries, displaying empathy, providing support, and managing their own emotional responses, even in challenging situations such as responding to patient aggression or coping with the death of a patient [3]. Importantly, EL is not limited to extreme events; it is present even in routine clinical tasks, such as inserting a cannula, where nurses must remain calm, compassionate, and reassuring [4]. EL in nursing has been found to play a vital role in delivering high-quality patient care, fostering trusting therapeutic relationships, and improving patient satisfaction [4–6]. However, engaging in EL can be both challenging and demanding. EL, particularly surface acting, has been associated with negative outcomes such as burnout, compassion fatigue, emotional dissonance, and anxiety [3, 4, 7–9]. On the contrary, deep acting has been shown to improve nurses' pride, self-efficacy, and personal accomplishment [4, 7, 10]. In addition, the EL of nurses has been linked to important work-related factors, including their intention to leave the profession, job attitudes, and the quality of patient care and satisfaction [7, 9, 11]. In the absence of adequate organizational support, nurses may rely more on surface acting more, which can lead to adverse effects on both individuals and healthcare organizations [7].

Based on the broaden-and-build theory of positive emotions of Fredrickson [12], positive affective states, such as those associated with job satisfaction, can expand individuals' psychological resources and foster their capacity for adaptive behaviors, including emotional regulation. From this perspective, nurses who experience higher job satisfaction may be more motivated and psychologically equipped to manage their emotional expressions in a healthier and more effective manner. Job satisfaction can influence how nurses respond to and manage the emotional demands of their role [4, 10]. Nurses with low job satisfaction may struggle with emotional strain, which can make it difficult for them to control and express their emotions appropriately in the workplace [13]. Evidence of the effects of nurses' job satisfaction on their EL is important to understand their interrelationship and develop interventions accordingly, as this can buffer the negative impact of EL on nurses' well-being and enhance nursing retention, which is essential in the context of global nursing shortages and the associated financial burden [7]. It can also predict the quality of care provided to patients because nurses' interaction with patients can be directly influenced by EL and job satisfaction. Nonetheless, most studies have examined job satisfaction as an outcome of EL [7, 10]; to the best of our knowledge, none have investigated it as a predictor of EL.

Although the EL concept in the nursing context was introduced in 1991, a literature review found only two studies conducted in Arab countries, one in Jordan [14] and the other in Saudi Arabia, involving nursing technicians in critical care units [15]. Nurses' utilization of EL can vary between countries according to social norms and religious and cultural factors, in addition to variations in healthcare systems and perceptions of nursing [10]. Arab countries are collective societies, where individuals are expected to show more warmth, hospitality, and care than in Western countries, where individuals contain their emotions and maintain distance [16].

Our study seeks to fill this gap in the literature by assessing the EL of nurses working in Saudi Arabia. The specific aims were to (1) assess nurses' EL and job satisfaction levels; (2) determine the association among EL, job satisfaction, and nurse-to-patient ratio; (3) determine whether job satisfaction predicts EL and; (4) assess the mean differences in EL according to nurses' personal and professional characteristics (age, gender, nationality, educational level, experience, department, shift, and nurse-to-patient ratio).

## 2. Methods

### 2.1. Theoretical Framework

**Figure 1:**
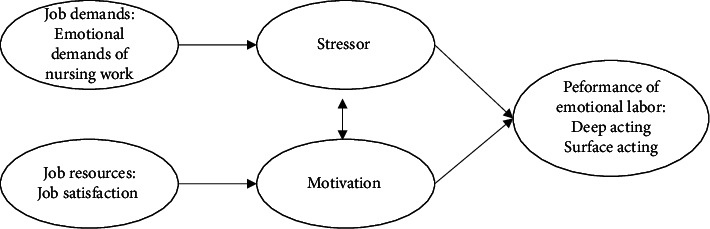
The job demand-resource (JD-R) model.

This study employed the job demand-resource (JD-R) model, which proposes that job demands (stressors) and job resources (motivational factors) interact in ways that can significantly influence employees' well-being and performance [17]. In our study, we examined how nurses' EL strategies were influenced by the emotional demands of nursing work as a job demand and by job satisfaction as a job resource (see Figure 1) [18].

### 2.2. Study Design and Setting

This was a cross-sectional, correlational, descriptive study. Data were collected from a public hospital in Riyadh, Saudi Arabia. The facility has a capacity of 2151 beds and is considered one of the most advanced and comprehensive healthcare institutions in the country. The study adhered to the Strengthening the Reporting of Observational Studies in Epidemiology (STROBE) guidelines.

### 2.3. Participants, Sample, and Sampling

A convenience sampling method was used to recruit registered bedside nurses who provide direct care to patients. Exclusion criteria included nursing students and interns, and nurses with only administrative tasks who do not care for patients, such as nurse managers and educators. G∗Power software 3.1 was used to determine the minimum sample size required to run the inferential analysis. Under a significance level of 0.05, a power of 0.80, and an effect size of 0.50, the minimum sample size needed for sufficient statistical power is 102 participants. To handle the possibility of missing data, the figure was increased by 10%, whereby 113 participants were deemed an adequate sample size for this study. The actual sample comprised 125 registered nurses working in the emergency room, intensive care unit, oncology ward, surgical ward, medical ward, hemodialysis, cardiac ward, and outpatient departments.

### 2.4. Data Collection

Data were collected from June to August 2024 through a hard-copy questionnaire and by research to ensure the eligibility of participants and to increase the response rate. Out of 140 distributed questionnaires, 125 were returned, resulting in an 89.2% response rate.

### 2.5. Measurements

The questionnaire began with a profile section to elicit the participants' information such as gender, age, qualification, and department. The second section was the Emotional Labor Scale for Nurses [19], which includes 17 items divided into four subscales: intensity (1 item); emotional control effort in the profession (7 items), which refers to nurses' work to understand the patient and maintain a therapeutic relationship; patient-focused emotional suppression (5 items), which detonates the actions of regulating and suppressing emotions such as fear; and emotional pretense by norms (4 items), which measures nurses' efforts to display a false facial expression and attitude in line with organizational and social expectations. The intensity subscale assesses time spent with patients in minutes, and the three latter subscales are rated on a five-point Likert scale. The scale score ranges from 16 to 80, where a higher score indicates a higher level of EL. The scale has a good reported validity and reliability, with Cronbach's alpha score of 0.81. In this study, Cronbach's *α* for the scale and subscales were 0.86, 0.89, 0.69, and 0.62, respectively. The last part measured job satisfaction using the Michigan Organizational Assessment Questionnaire Job Satisfaction scale [20], which consists of three items scored on a five-point Likert scale. The score ranges from 3 to 15, where a higher score indicates higher job satisfaction. The scales have satisfactorily reported validity and reliability, and Cronbach's *α* in this study was 0.78. Data were collected in English, as it is the official language used in the hospital.

### 2.6. Data Analysis

The Statistical Package for the Social Sciences software (SPSS version 30) was used to manage and analyze the data. The participants' information and the EL of nurses and job satisfaction were reported in means, standard deviations, numbers, percentages, and ranges. Person's product moment correlations were used to assess the association between the scale and subscales of the EL, job satisfaction, and nurse-to-patient ratio. Simple linear regression was used to determine whether job satisfaction predicts EL while controlling for participants' characteristics. These variables were chosen based on the significant correlation between EL and job satisfaction, alongside the JD-R model and support from previous empirical studies. Independent-sample *t*-tests were used to assess the mean differences in the participants' EL according to their personal and professional characteristics. Due to variations in group sizes, departments were categorized into critical units (intensive care, hemodialysis, oncology, and emergency) and noncritical units (medical, surgical, and outpatient). The significance level was set at *p* < 0.05.

### 2.7. Ethical Considerations

This study was approved by the Institutional Review Board of King Abdullah International Medical Research Center (KAIMRC). This study adhered to the Declaration of Helsinki and the Belmont Report. The target population received a consent form stating the purpose of the study, its risk and benefits, measures to maintain privacy and confidentiality, and the eligibility criteria to participate in the study. In addition, they were informed about the right of voluntary participation and assured that they could withdraw from the study at any time without any potential consequences. Participants' anonymity was assured; no names or identifiers were collected, and the data are only reported in aggregate form. Participants were asked to sign the consent form if they wished to participate. All data in both hard and soft forms were stored securely and accessed only by the researcher.

## 3. Result

Table 1 presents the participants' demographic data. The mean age of participants was *M* = 35.30 (SD ± 6.97); the majority were female (85.5%), and approximately two-thirds were expatriates (66.8%). Most participants held a bachelor's degree (68.0%) and had three or more years of experience in nursing (51.6%). Most participants were from the medical unit (21.6%) and worked shifts (80.8%). The mean nurse-to-patient ratio was *M* = 8.08 (SD ± 13.49).

Table 2 shows the mean scores for the EL and job satisfaction of nurses. Their EL level was high (*M* = 58.04, SD ± 9.44). The intensity mean score was *M* = 17.39 (SD ± 16.56), indicating that most of the participants spent around 17 min interacting with patients. The level of emotional control effort in the profession was high (*M* = 28.65, SD ± 5.09), indicating that the majority of the participants worked to understand the patient and maintain a therapeutic relationship with them. The level of patient-focused emotional suppression was moderate (*M* = 16.15, SD ± 3.82), indicating that some of the participants undertook to regulate and suppress their emotions. The level of emotional pretense by norms was moderate (*M* = 13.23, SD ± 2.89), which shows that some of the participants displayed false facial expressions and attitudes in line with organizational and social expectations. Lastly, the level of job satisfaction was moderate (*M* = 10.27, SD ± 2.61), indicating that the participants felt somewhat satisfied with their job.

Pearson's correlations for the associations among EL, job satisfaction, and nurse-to-patient ratio are presented in Table 3. The findings revealed strong positive relationships between EL and emotional control effort in the profession (*r* = 0.815, *p* < 0.001), patient-focused emotional suppression (*r* = 0.792, *p* < 0.001), and emotional pretense by norms (*r* = 0.780, *p* < 0.001, respectively). Furthermore, a significant correlation was found between job satisfaction and both emotional control effort in the profession and EL (*r* = 0.258, *p* = 0.004; *r* = 0.220, *p* = 0.014, respectively). There was also a significant correlation between the nurse-to-patient ratio and emotional pretense by norms (*r* = 0.179, *p*=0.046).

The results of the simple linear regression indicated a significant relationship (*F* [1, 123] = 6.235, *p*=0.014, *R*^2^ = 0.041), indicating that job satisfaction (*β* = 0.22, *p*=0.014) statistically significantly predicted the EL of nurses (Table 4), such that nurses with greater job satisfaction had greater EL.

The independent-sample *t*-tests were used to assess the mean differences in EL for nurses according to the demographic factors (Table 5). For nationality, the results revealed that local nurses had a higher EL intensity (*M* = 22.03, SD ± 21.23, *p* = 0.037, *d* = 0.41) and emotional pretense by norms (*M* = 13.87, SD ± 3.14, *p* = 0.048, *d* = 0.32) than expatriate nurses. Married participants showed greater emotional control in the profession (*M* = 29.62, SD ± 4.61, *p* = 0.022, *d* = 0.36) than those who were single. Participants who had 3 years of experience or less had higher emotional pretense by norms (*M* = 13.71, SD ± 3, *p* = 0.039, *d* = 0.31) and EL (*M* = 59.75, SD ± 9.01, *p* = 0.024, *d* = 0.35) than those with more than 3 years of experience. Furthermore, those who worked shifts had higher patient-focused emotional suppression (*M* = 16.43, SD ± 3.94, *p* = 0.045, *d* = 0.38) than those who did not. There was a strong, significant difference in EL intensity by department, such that nurses who worked in the critical units had higher EL intensity (*M* = 20.53, SD ± 19.23, *p* = 0.006, *d* = 0.43) than those who worked in noncritical areas.

## 4. Discussion

This study examined the EL level of nurses and the impact of job satisfaction. Through the lens of the JD-R model, our findings offer a more nuanced understanding of how job demands and resources interact in a high-stakes, emotionally intensive profession. Consistent with the JD-R model, the high levels of EL reported by nurses reflect the significant emotional demands of their work. While the majority of nurses were engaged in deep acting, genuinely caring for their patients, some frequently relied on surface acting, suppressing or faking emotions, to maintain professionalism and meet organizational expectations. This form of emotional regulation represents a clear job demand that can deplete psychological energy over time and potentially lead to burnout if not buffered by adequate resources.

The study findings indicate that the EL of the nurses was high. When EL is integrated into nursing work, it helps nurses build and maintain trusting relationships with patients, rather than just completing tasks [21]. The form most reported by participants was honest feelings of compassion and empathy toward patients instead of suppressing or faking emotions. This is supported by reviews that have reported that nurses often do not fake their emotions but genuinely feel and express care [7, 22]. These caring emotions are a source of joy, fulfillment, pride, professionalism, and accomplishment for nurses that reflect positively on their well-being and job satisfaction [7, 23], which might explain the adequate level of nurses' job satisfaction despite the emotional demands of the profession. Although the EL of nurses, namely, deep acting, can be linked to better outcomes for nurses, patients, and the organization, it has been exploited by organizations. One study [24] argued that organizations expect nurses to show care and manage their emotions, without providing support or training. Chronic and prolonged emotional engagement and demands without proper support can result in negative outcomes by draining nurses' emotions. A review of pediatric oncology nurses' EL found that over time, these nurses tend to shift from deep acting to surface acting to cope with emotional exhaustion, which altered their caring behaviors and work performance [4]. Some nurses avoided emotional engagement and challenging situations by reducing time spent in patients' rooms, steering clear of dying patients, or resorting to absenteeism.

According to the findings, nurses sometimes had to show fake emotions and/or suppress and regulate their feelings when caring for patients, similar to earlier findings [7, 22]. These EL strategies can help nurses manage patients, build good rapport, and complete their tasks. However, consideration should be given to the fact that faking emotions and facial expressions requires emotional, physical, and mental efforts, resulting in several adverse outcomes for the nurses and the organization [7, 9]. Surface acting has been associated negatively with nurses' health, resulting in a range of occupational, emotional, and physical issues [3, 7, 22, 23, 25]. Nevertheless, the findings of the “Emotional Pretense by Norms” subscale should be interpreted with caution, as its Cronbach's alpha (0.62) falls below the commonly accepted threshold for reliability (0.70). Future research should consider validating this subscale across the same cultural group to improve its reliability.

Job satisfaction emerged as a critical resource that moderated the impact of these emotional demands. Although nurses reported only moderate levels of job satisfaction, it was strongly correlated with their emotional control efforts and with overall EL, in line with previous studies [7, 26]. Several studies have indicated that nurses' deep acting is positively correlated with nurses' professional identity and job satisfaction [3, 7, 13, 23, 27]. Similarly, this study found a significant correlation between nurses' emotional control effort in the profession and their job satisfaction. However, in contrast, a study in Turkey found a negative correlation among nurses' EL, deep acting, and job satisfaction [26]. Possible explanations are differences in the participants' characteristics and utilization of EL and in the instrument used. Researchers have highlighted a significant negative correlation between surface acting and job satisfaction [7, 9, 22, 27, 28]. In this study, a negative but insignificant association was found between these variables; however, the nurses' emotional pretense by norms was associated with the nurse-to-patient ratio, such that as nurses cared for more patients, they had to fake their emotions and actions to a greater extent to meet organizational and social expectations.

Despite the weaker correlation with EL, job satisfaction still played a meaningful buffering role. The participants' job satisfaction significantly influenced their EL, accounting for 4.1% of the variance, aligning with the JD-R model's core proposition that job resources can mitigate strain and enhance performance. Nurses who reported higher satisfaction appeared to be better equipped to manage their emotional expressions without experiencing as much psychological strain. Although no studies to date have examined job satisfaction as a predictor of EL among nurses, similar findings have been reported in research involving travel agency employees [29]. The results indicated that job satisfaction significantly influenced deep acting (*β* = 0.534, *p* < 0.001, *R*^2^ = 0.298) and had a negative effect on surface acting (*β* = −0.230, *p* < 0.001, *R*^2^ = 0.090). These findings suggest that job satisfaction may function as a driver of emotional regulation strategies rather than merely serving as an outcome. The job satisfaction of nurses can improve their resilience and management of the emotional demands of work and buffer its impact on them [25]. In addition, it can impact how nurses utilize EL strategies. As their perceived job satisfaction increases, nurses use deep acting more than surface acting, which is associated positively with better individual and organizational outcomes [7, 10, 23]. In contrast, nurses with low job satisfaction tend to use surface acting more frequently, which can further decrease their job satisfaction and negatively impact their well-being and performance [7]. However, the modest proportion in our study underscores the complex and multifaceted nature of EL, which is likely shaped by a range of individual, organizational, and contextual factors.

Our findings indicated variations in EL level and utilization between the participants. The local nurses displayed higher EL intensity and emotional pretense by norms than expatriate nurses. Islam, which is the religion in Saudi Arabia, encourages Muslims to show compassion, care, and patience, which could influence how local nurses show emotions as part of their religious beliefs [30]. A study in Iran indicated that nurses greeted patients with a smiling face to demonstrate love and compassion, in line with their Islamic religious belief [31]. Furthermore, Halligan [30] argues that the nurses' experience of EL is influenced by patients' religious beliefs and cultural traditions. By sharing the same language and culture, local nurses could face a greater emotional need to engage with patients to meet cultural norms and expectations, unlike expatriate nurses, who might be less familiar with the local language and cultural and emotional norms [32]. Hence, Saudi nurses might have interacted longer with patients and changed their facial expressions according to cultural and social norms more than expatriate nurses [33]. Furthermore, the nursing workforce in Saudi Arabia has relied on expatriate nurses for years, as more than half of the nursing workforce is expatriates [34], which could impose greater pressure on Saudi nurses to display the appropriate emotional and facial expressions in interactions to conform and demonstrate their trustworthiness to society [35, 36]. Nurses with 3 years of experience or less had higher EL and emotional pretense by norms, which was reported in an earlier review as well [4]. Novice nurses might find it difficult to master and manage EL, unlike senior nurses, who are accustomed to and adapted to emotions at work. Nurses working in shifts in our study and in Turkey [26] had to regulate and suppress their emotions more than those who did not, likely because they were exhausted from changing sleep schedules and the psychological experience of shift work [37]. Conversely, no significant variation by the department was found in the overall EL and its strategies, though EL intensity was higher in the critical units. Patients in these areas are often critically ill and require certain tasks and procedures that might take longer. Additionally, nurses in noncritical units might have had more patients, causing them to spend less time with patients to accommodate all the care required for all their assigned patients within the shifts [38].

## 5. Implications

The study findings afford valuable insights, not only in Saudi Arabia and countries with similar norms but also internationally, as most of the participants were expatriate nurses. The findings underscore the high level of nurses' EL and the need to address it at the individual and organizational levels, as it is essential for nurses' well-being, patient quality care, and the organization's performance and profit. Policymakers and healthcare managers should prioritize enhancing nurses' competencies in applying EL strategies and managing its impact, particularly for novice nurses, by incorporating relevant training into nursing programs. Furthermore, targeted interventions to strengthen emotional intelligence, self-efficacy, and resilience are essential for improving nurses' emotional regulation and coping skills. These can include ongoing professional development, promoting autonomy, debriefing practices, fostering a culture of empowerment, and implementing supportive leadership and mentorship practices [7, 39]. At the organizational level, several supportive interventions should be implemented to minimize and manage the adverse effects of EL. These include providing access to professional consultation services, implementing strategies to enhance nurses' job satisfaction, formally recognizing and rewarding emotional work, enforcing policies to maintain recommended nurse-to-patient ratios, and ensuring reasonable workloads through adequate staffing [25, 40]. The EL and job satisfaction of nurses are interconnected, and as job satisfaction can influence EL, interventions to improve either can in fact enhance both. Introducing EL into the nursing curriculum can assist nursing students in acquiring the skill of using EL and its strategies from an early stage in their profession.

## 6. Limitations and Future Research Directions

This is the first study to empirically examine the relationships between the EL of nurses and job satisfaction in Saudi Arabia. Despite its novelty, there are several limitations that affect the generalizability of our findings to broader nursing populations. First, although the study provides valuable insights, it relied on convenience sampling and involved a relatively small number of nurses from a single institution. Second, data were collected exclusively from a public hospital, which may further limit the applicability of the results. Private hospitals often differ in terms of resource availability, staffing structures, and patient demographic factors that could significantly influence outcomes. Additionally, healthcare systems vary widely across countries in terms of organization, policy, and clinical practice. Future research that includes a more diverse sample from multiple hospital types and international settings would help enhance the external validity and robustness of these findings. In addition, the cross-sectional design limits the confirmation of the causal association between EL and job satisfaction and examines changes in EL over time. Lastly, using a self-report measure might result in self-selection, recall, and social desirability biases, which could influence the findings.

It is recommended that the study be replicated in both the private and public sectors, with a larger number of nurses from a wider variety of wards. Further studies are needed regarding the personal and organizational factors influencing nurses' EL as well as the impact of EL on nurses' physical and mental health, performance, turnover intention, and patient satisfaction using longitudinal studies and multisource data. These data can assist in further understanding the impact of nurses' EL on a wider scale and inform policy and interventions accordingly. Future research in Saudi Arabia should build on this study by exploring nurses' EL using a mixed-methods approach. Interviewing nurses in addition to measuring their EL can provide greater insight into local and expatriate nurses' perspectives and experiences of EL and the religious and cultural influences upon them.

## 7. Conclusion

This study clarified the EL level and strategies among nurses in Saudi Arabia and revealed the impact of job satisfaction on nurses' EL, which is an area for which limited evidence is available. Our findings suggest the need to recognize and address the emotional requirements of nursing work, for while most nurses were engaged emotionally when caring for patients, some had to fake or suppress their emotions. Among our participants, the presentation of emotions according to norms was higher among local and novice nurses, which could exert a negative impact on nurses' well-being, such as emotional dissonance and burnout. We recommend enhancing nurses' job satisfaction as key to the proper utilization of EL and management of its adverse effects.

## Figures and Tables

**Table 1:** Participants' information (*N* = 125).

Variable	*N* (%) or *M*(SD)
Age	35.30 (6.97)
Gender	
Female	106 (85.5)
Male	18 (14.5)
Nationality	
Local	39 (31.2)
Expatriate	86 (68.8)
Marital status	
Married	62 (50.8)
Single	60 (49.2)
Educational level	
Diploma	28 (22.4)
Bachelors	85 (68.0)
Master or higher	12 (9.6)
Experience in nursing	
Three years and less	60 (48.4)
More than 3 years	64 (51.6)
Department	
Medical unit	27 (21.6)
Surgical unit	20 (16.0)
Emergency room	17 (13.6)
Hemodialysis	16 (12.8)
Oncology unit	14 (11.2)
Cardiac unit	13 (10.4)
Intensive care unit	10 (8.0)
Outpatient departments	8 (6.4)
Shift	
Yes	101 (80.8)
No	24 (19.2)
Nurse-to-patient ratio	8.08 (13.49)

**Table 2:** Emotional labor and job satisfaction scores (*N* = 125).

Variables	*M*	SD±	Range
Emotional labor scale	58.04	9.44	16–80
Intensity			
A typical interaction I have with a patient takes about minutes	17.39	16.56	1–100
Emotional control effort in profession	28.65	5.09	7–35
I try to be kind to patients genuinely from my heart	4.41	0.89	1–5
I try to change my emotions to the positive forms patients expect	4.20	0.92	1–5
I try to adjust my emotions and attitude depending on patients' emotion change	3.83	1.06	1–5
I express my emotion to maintain continuous rapport with patients	3.78	1.02	1–5
I manage my expression and way of speaking with professional attitude to maintain patients' trust	4.31	0.83	1–5
I try to understand different circumstances between doctors and patients	4.16	0.83	1–5
I try to overcome emotionally difficult situations with a sense of vocation as a nurse	3.97	0.879	1–5
Patient-focused emotional suppression	16.15	3.82	5–25
I suppress my anger when patients' words and behaviors are unfair	3.64	1.04	1–5
I tolerate verbally and nonverbally violent behavior from patients. Even though I feel scared	3.05	1.19	1–5
I tolerate patients expressing negative emotion about medical staff or other departments to me	2.65	1.18	1–5
I control my mind, thinking that patience is a virtue	3.91	0.86	1–5
I tolerate unfair treatment to maintain a good work atmosphere on the ward	2.94	1.34	1–5
Emotional pretense by norms	13.23	2.89	4–20
I exaggerate expressions of interest in patients	2.90	1.02	1–5
I pretend to feel what I don't actually feel when I deal with patients (e.g., empathy, interest, friendliness, delight, etc.)	2.90	1.19	1–5
I consciously control my facial expression. Attitude. And way of speaking when interacting with patients	3.70	1.04	1–5
Although patients make me emotionally uncomfortable. I treat them with positive facial expression and attitude changed instantly	3.75	0.94	1–5
Job satisfaction	10.27	2.61	3–15

**Table 3:** Correlation between emotional labor, job satisfaction, and patient ratio (*N* = 125).

**Variables**		**1**	**2**	**3**	**4**	**5**	**6**	**7**

1. Intensity	*r*	1						
	*p*							

2. Emotional control effort in profession	*r*	−0.086	1					
	*p*	0.340						

3. Patient-focused emotional suppression	*r*	0.045	0.366	1				
	*p*	0.616	< 0.001^∗∗^					

4. Emotional pretense by norms	*r*	−0.036	0.413	0.618	1			
	*p*	0.688	< 0.001^∗∗^	< 0.001^∗∗^				

5. Emotional labor scale for nurses	*r*	−0.039	0.815	0.792	0.780	1		
	*p*	0.664	< 0.001^∗∗^	< 0.001^∗∗^	< 0.001^∗∗^			

6. Job satisfaction	*r*	−0.043	0.258	0.146	−0.069	0.220	1	
	*p*	0.631	0.004^∗^	0.104	0.444	0.014^∗^		

7. Nurse-to-patient ratio	*r*	−0.024	0.086	0.073	0.179	0.127	0.102	1
	*p*	0.780	0.322	0.372	0.046^∗^	0.129	0.281	

^∗^ *p* < 0.05.

^∗∗^ *p* < 0.001.

**Table 4:** Linear regression analysis of job satisfaction and emotional labor scale for nurses (*N* = 124).

Outcomes	Emotional labor scale for nurses						
	*B* ^a^	SE *B*	*β* ^b^	*t*	*p*	95% CI	
						Lower	Upper
Job satisfaction	0.792	0.317	0.220	2.497	0.014	0.164	1.420
Model summary	(*F* [1, 123] = 6.235. *p*=0.014. *R*^2^ = 0.041)						

*Note:* A simple linear regression model was used. Predictor: Job satisfaction. Outcome variable: Emotional labor; *R*^2^: adjusted *R*-squared value. *p* < 0.05.

^a^B coefficient.

^b^Beta standardized coefficient.

**Table 5:** Mean differences in emotional labor scale for nurses according to the demographic factors (*N* = 125).

Demographic factors	*N*	Intensity				Emotional control effort in profession				Patient-focused emotional suppression				Emotional pretense by norms				Emotional labor scale for nurses			
		*M*(SD)	*t* *p*	95% CI		*M*(SD)	*t* *p*	95% CI		*M*(SD)	*t* *p*	95% CI		*M*(SD)	*t* *p*	95% CI		*M*(SD)	*t* *p*	95% CI	
Categories				LPCI	UPCI			LPCI	UPCI			LPCI	UPCI			LPCI	UPCI			LPCI	UPCI
Nationality																					
Local	39	22.03 (21.23)	1.819 0.037^∗^	0.49	12.98	27.97 (4.61)	1.008 0.158	−2.93	0.95	16.46 (3.56)	0.607 0.272	−1.01	1.91	13.87 (3.14)	1.674 0.048^∗^	−0.17	2.02	58.30 (9.47)	0.213 0.416	−3.23	4.01
Expatriate	86	15.29 (13.58)		−0.69	14.16	28.96 (5.29)		−2.84	0.86	16.01 (3.95)		−0.96	1.86	12.94 (2.74)		−0.23	2.09	57.91 (9.48)		−3.25	4.03
Marital status																					
Married	62	17.34 (16.31)	0.097 0.461	−6.32	5.73	29.62 (4.61)	2.030 0.022^∗^	0.04	3.69	16.32 (4.01)	0.539 0.296	−1.00	1.76	13.26 (2.73)	0.093 0.463	−1.00	1.09	59.21 (8.70)	1.335 0.092	−1.10	5.69
Single	60	17.64 (17.29)		−6.32	5.73	27.75 (5.51)		0.04	3.69	15.95 (3.70)		−1.00	1.76	13.21 (3.10)		−1.00	1.09	56.91 (10.22)		−1.10	5.70
Experience																					
Three years and less	60	16.47 (13.91)	0.637 0.263	−7.83	4.01	29.35 (4.50)	1.531 0.064	−0.40	3.20	16.68 (3.89)	1.518 0.066	−0.31	2.40	13.71 (3.00)	1.775 0.039^∗^	−0.10	1.94	59.75 (9.01)	1.996 0.024^∗^	0.02	6.69
More than 3 years	64	18.38 (18.87)		−7.78	3.96	27.95 (5.56)		−0.39	3.19	15.64 (3.75)		−0.31	2.40	12.79 (2.76)		−0.10	1.94	56.39 (9.68)		0.03	6.68
Shift																					
Yes	101	17.83 (16.83)	0.607 0.272	−5.18	9.76	28.36 (5.45)	1.308 0.097	−3.79	0.77	16.43 (3.94)	1.713 0.045^∗^	−0.22	3.18	13.38 (3.02)	1.222 0.112	−0.49	2.10	58.18 (10.16)	0.358 0.360	−3.48	5.03
No	24	15.54 (15.62)		−5.01	9.59	29.87 (2.93)		−3.12	0.10	14.95 (3.08)		−0.01	2.97	12.58 (2.22)		−0.29	1.89	57.41 (5.56)		−2.26	3.80
Department																					
Critical units	70	20.53 (19.23)	2.581 0.006^∗∗^	1.33	12.92	28.81 (5.05)	0.391 0.348	−1.46	2.18	16.31 (4.06)	0.533 0.297	−1.00	1.73	13.10 (3.24)	0.573 0.284	−1.33	0.73	58.23 (10.01)	0.251 0.401	−2.95	3.80
Noncritical units	55	13.40 (11.35)		1.65	12.59	28.45 (5.19)		−1.47	2.19	15.95 (3.54)		−0.97	1.71	13.40 (2.42)		−1.30	0.70	57.80 (8.75)		−2.89	3.75

^∗^ *p* < 0.05.

^∗∗^ *p* < 0.01.

## Data Availability

The data that support the findings of this study are available on request from the corresponding author. The data are not publicly available due to privacy or ethical restrictions.
